# Beyond Single-Pathogen Models: Understanding Mixed Infections Involving Phytoplasmas and Other Plant Pathogens

**DOI:** 10.3390/plants14132049

**Published:** 2025-07-04

**Authors:** Shao-Shuai Yu, Wei Wei

**Affiliations:** 1 Coconut Research Institute, Chinese Academy of Tropical Agricultural Sciences, Wenchang 571339, China; 2 Molecular Plant Pathology Laboratory, Beltsville Agricultural Research Center, Agricultural Research Service, United States Department of Agriculture, Beltsville, MD 20705, USA

**Keywords:** ‘one pathogen, one disease’ concept, plant pathobiome paradigm, co-infection, unculturable plant pathogen, plant disease prevention

## Abstract

Phytoplasmas are wall-less, phloem-restricted bacteria responsible for numerous significant plant diseases worldwide. An increasing body of evidence indicates that phytoplasmas can coexist with other pathogens in mixed infections, including various 16Sr group phytoplasmas, ‘Candidatus Liberibacter’ species, viruses, spiroplasmas, fungi, and other difficult-to-culture phloem-limited bacteria. These interactions challenge established views regarding the causes, detection, and management of plant diseases. This review consolidates existing knowledge on the diversity and epidemiology of phytoplasma-related mixed infections, with a particular emphasis on documented co-infections across various host plants and regions, especially in tropical and subtropical areas. Mixed infections affect disease severity, symptom expression, vector behavior, and pathogen dissemination, highlighting the limitations of pathogen-specific diagnostic and control strategies. The necessity for tools to detect multiple pathogens, enhanced understanding of pathogen–pathogen and host–pathogen interactions, and comprehensive surveillance systems is emphasized. Ultimately, breeding for resistance must consider the complexities of natural co-infections to ensure effective protection of crops. Addressing the challenges presented by phytoplasma-related mixed infections is crucial for developing resilient and sustainable plant health strategies in the face of increasing ecological and agricultural pressures.

## 1. Introduction

Plant disease epidemics are increasingly complex due to the prevalence of mixed infections, in which multiple pathogens infect the same host simultaneously [1]. This marks a shift from the traditional ‘one pathogen, one disease’ paradigm in plant pathology, where a single pathogen is linked to specific symptoms and disease outcomes. While this model has historically guided diagnosis and management, it fails to account for evidence that plants often harbor multiple pathogens that interact unpredictably [2]. Once thought to be rare, mixed infections are now recognized as common in both natural and agricultural ecosystems [3]. This change is a result of various global factors, including climatic shifts, intensified monoculture practices, increased international trade, and the movement of insect vectors across regions. These conditions create environments that support the coexistence of diverse pathogens within a single host, complicating disease diagnosis, epidemiology, and management [4,5,6,7].

Phytoplasmas are among the pathogens involved in mixed infections. They are a group of phloem-limited, wall-less bacteria classified under the class Mollicutes [8,9,10,11]. Initially identified by Doi and colleagues in 1967 as mycoplasma-like organisms (MLOs) [12], phytoplasmas have gained recognition for their impact on various (nearly 1000) plant species, influencing their functionality and causing substantial economic repercussions [13,14,15,16,17,18]. The term ‘phytoplasma’ was officially adopted in 1994 to clarify their taxonomic classification. These pathogens are now categorized under the provisional genus ‘*Candidatus* Phytoplasma’, with recent guidelines improving species-level descriptions [8,19,20].

Phytoplasmas exhibit several unique biological characteristics that set them apart from other bacterial pathogens. A key feature is their lack of a rigid cell wall, which contributes to their pleomorphic morphology. Additionally, they cannot be cultured in vitro, making laboratory studies particularly challenging [12,14]. These pathogens inhabit only the phloem tissues of infected plants and require phloem-feeding insect vectors, such as leafhoppers and planthoppers, for their transmission. Once they enter the host plant, phytoplasmas secrete effector proteins that disrupt essential cellular functions, including hormone signaling, meristem development, and chloroplast integrity. These changes result in a variety of distinctive symptoms, including witches’ broom, phyllody, virescence, stunted growth, and reduced photosynthetic capacity [13,14,21,22].

Despite the biological distinctiveness and agricultural importance of phytoplasmas, their role in mixed infections remains poorly understood. Co-infections involving phytoplasmas and other pathogens, such as viruses, fungi, spiroplasmas, and other phloem-associated bacteria, have been increasingly reported across diverse agroecosystems [9,14,23,24,25,26,27,28,29,30,31,32,33,34,35,36,37,38]. These interactions can involve multiple strains or species within the same host, sometimes even within the same tissue, raising questions about pathogen competition, cooperation, and shared vector dynamics. Such complex interactions may influence symptom expression, disease progression, vector behavior, and pathogen transmission efficiency, often in ways that are difficult to predict or generalize.

The diagnostic and management implications of these co-infections are significant. The presence of multiple pathogens can obscure typical disease symptoms, delay accurate diagnosis, and reduce the effectiveness of targeted control strategies. For example, resistance developed against one pathogen may prove ineffective in the presence of additional pathogens, while pesticide applications may not fully address the spectrum of causal agents. Moreover, the inability to culture phytoplasmas in vitro hampers experimental investigations of their interactions with co-infecting organisms, necessitating the use of advanced molecular tools for detection and analysis.

These challenges are not unique to plant pathology. Similar complexities arising from co-infections are well-documented in other biological systems, including human and animal health [3,39,40]. In humans, interactions between commensal and pathogenic microbes influence disease severity, immune responses, and treatment outcomes [39,41,42]. In aquaculture, simultaneous infections by viruses, bacteria, and parasites intensify disease impacts and compromise host resilience [40,43,44,45,46]. These parallels highlight the broader biological relevance of co-infection phenomena and the need for cross-disciplinary insights into mixed pathogen dynamics.

In plant systems, mixed infections involving phytoplasmas represent an urgent and understudied area. They reveal critical gaps in our understanding of host–pathogen interactions, diagnostic precision, and disease ecology. Although molecular tools such as quantitative PCR, high-throughput sequencing, and metagenomics have improved the detection of multiple pathogens in complex samples [3,33,35,36,47,48,49], significant barriers remain in linking this data to biological functions, ecological impact, and management outcomes.

This review aims to provide a comprehensive synthesis of current knowledge on mixed infections involving phytoplasmas and other plant pathogens. It explores the biological mechanisms underlying pathogen interactions, the epidemiological consequences of co-infection, the technical and diagnostic challenges presented by these complex disease systems, and the implications for effective disease surveillance, resistance breeding, and integrated management strategies. This review aims to highlight the role of phytoplasmas in mixed infections by integrating insights from molecular biology, ecology, and agricultural practices while also offering ideas for developing more resilient and knowledge-based plant health management strategies.

## 2. Mixed Infections of Phytoplasmas from Distinct 16Sr Groups

Phytoplasmas are classified into ribosomal (16Sr) groups based on sequence similarity within the 16S rRNA gene. This system, developed through restriction fragment length polymorphism (RFLP) and sequence analysis, provides a framework for identifying phytoplasma strains and understanding their evolutionary relationships [50,51,52]. To date, 37 16Sr groups and over 100 subgroups have been described, each associated with specific plant hosts, disease symptoms, and geographic regions [8]. This genetic diversity has important implications for host specificity, symptom variability, and transmission dynamics.

While many studies of phytoplasma-associated diseases have traditionally focused on single-pathogen infections, an increasing number of reports now highlight the occurrence of mixed infections involving phytoplasmas from different 16Sr groups within the same host plant [25,26,36]. These co-infections suggest a higher level of ecological and epidemiological complexity than previously assumed. They may arise through the sequential or simultaneous inoculation of plants by vectors carrying different phytoplasma strains, especially in areas with overlapping distributions of host plants and insect vectors.

Mixed infections have the potential to influence disease expression in unpredictable ways. They may enhance symptom severity, alter host–pathogen interactions, and contribute to the emergence of novel disease phenotypes. Moreover, the coexistence of multiple phytoplasma strains within the same vascular system may facilitate genetic exchange, although evidence for horizontal gene transfer in phytoplasmas remains limited.

### 2.1. Case Studies in Jujube and Elaeocarpus spp.

Several case studies provide evidence of mixed infections by phytoplasmas from different 16Sr groups under natural conditions. One of the earliest well-documented examples comes from South Korea, where jujube (*Ziziphus jujuba*) trees exhibiting witches’ broom symptoms, such as chlorosis, shortened internodes, reduced leaf size, and abnormal shoot proliferation, were found to be co-infected with phytoplasmas from both the 16SrI and 16SrV groups. Detection was achieved through nested PCR amplification of the 16S rRNA gene using the primer sets P1/P7 and R16F2n/R16R2 [25].

A similar instance was observed in Henan Province, China, where Sun et al. [26] identified co-infection by 16SrI and 16SrV phytoplasmas in jujube trees through 16S rRNA gene sequence analysis. These findings, consistent across different geographic locations, reinforce the notion that mixed phytoplasma infections are not isolated anomalies but may occur wherever multiple phytoplasma-infected hosts and compatible insect vectors coexist (Table 1).

A recent study by Lee et al. [36] investigated Elaeocarpus sylvestris trees exhibiting decline symptoms on Jeju Island, South Korea. The study uncovered co-infection with phytoplasmas from the 16SrI and 16SrXXXII groups through advanced molecular techniques [36] (Table 1). Employing multilocus sequence analysis (MLSA), the study focused on the ribosomal protein (*rp*) operon, elongation factor Tu gene (*tuf*), and *secA* gene, moving beyond the traditional focus on the 16S rRNA gene found in earlier studies. This methodology allowed for high-resolution identification of multiple phytoplasma strains within a single host, demonstrating the utility of MLSA in complex disease situations.

**Table 1:** Mixed infections of multiple phytoplasma 16Sr groups in various hosts across regions.

Phytoplasmas/16Sr Groups	Phytoplasmas Involving Mixed Infections	Hosts	Symptoms	Locations	References
16SrI	16SrV	Jujube	witches’ broom	Korea	[25]
16SrI	16SrV	Jujube	witches’ broom	Henan, China	[26]
16SrI	16SrXXXII	*Elaeocarpus sylvestris*	decline	Jeju Island, Korea	[36]
16SrI-B	16SrII-D	Rose	phyllody, flower malformation, little leaf, flat stem	Delhi, Manipur, Uttar Pradesh, and Maharashtra in India	[53]
16SrI-X	16SrII-N	Papaya	bunchy top	Cuba	[27]
16SrI-B	16SrI-G, 16SrV	Grapevine	*flavescence dorée*	Piemonte, Italy	[54]
16SrI-B	16SrI-C, 16SrVII-A, 16SrXII-A	Grapevine	yellows	Chile	[55]
16SrX-B	16SrXII-A	Grapevine	yellows	Serbia	[56]
16SrI ‘*Ca.* Phytoplasma asteris’	16SrVII ‘*Ca.* Phytoplasma fraxini’	Potato	foliage yellowing, curling on upper leaf side, purple margins	Cundinamarca, Colombia	[57]
16SrI-B	16SrXII-A	Lily	chlorosis, leaf necrosis, malformation	Poland	[58]
16SrI-C	16SrIII-B	Clover	phyllody, dwarf	Lithuania	[59]
16SrII-D ‘*Ca.* Phytoplasma australasiae’	16SrXXIX ‘*Ca.* Phytoplasma omanense’	*Diospyros kaki*	yellowing, reddening, die-back	Mehriz (Yazd province), Iran	[60]
16SrI-B	16SrI-X	*Melia dubia*	mosaic, leaf curl, stunted growth	India	[38]

The identification of mixed phytoplasma infections presents significant diagnostic hurdles. Conventional PCR methods that focus on the 16S rRNA gene may overlook minority strains or yield unclear results due to the presence of dominant strains. As noted by Lee et al. [36], MLSA incorporates multiple housekeeping and protein-coding genes, presenting a comprehensive and precise approach for analyzing mixed infections. Furthermore, MLSA establishes phylogenetic relationships among phytoplasmas and distinguishes closely affiliated strains, which is vital for accurate diagnosis and epidemiological surveillance. It also enhances the resolution for atypical or novel strains that are challenging to assess using 16S rRNA methodologies. Implementing these techniques is crucial for tracking phytoplasma diversity, particularly in regions prone to co-infections.

### 2.2. Host and Vector Ecology in Facilitating Mixed Infections

Mixed infections involving multiple 16Sr groups are heavily influenced by host distribution and vector ecology. In regions where susceptible host plants are cultivated in proximity, the likelihood of cross-infection by phytoplasma-carrying vectors increases. In several regions of China, paulownia and jujube trees, recognized hosts for 16SrI and 16SrV phytoplasmas, are widely cultivated. It has been suggested that leafhopper vectors, which can feed on both plant species, act as bridges for the cross-species transmission of phytoplasmas. This could result in the introduction of 16SrI phytoplasmas into jujube trees and vice versa [26,61]. Such ecological overlaps create a favorable environment for dual colonization of host phloem tissues, increasing the risk of mixed infections. Furthermore, the presence of multiple phytoplasmas in the same vector population could enhance transmission efficiency and pathogen diversity, potentially facilitating the evolution of more virulent or adaptable phytoplasma strains.

### 2.3. Implications for Disease Expression, Detection, and Evolution

The occurrence of mixed phytoplasma infections has significant implications for the management of plant health. First, co-infection may result in atypical or overlapping symptoms, making field diagnosis unreliable [25,26,36]. Second, these infections complicate molecular detection, especially when single-locus methods are used [25,26,36]. There is also a possibility that phytoplasmas in mixed infections may interact synergistically, resulting in enhanced disease severity or accelerated host decline. Furthermore, the presence of various phytoplasma strains within a single host plant poses risks of genetic recombination or horizontal gene transfer, which may result in the development of new strains that possess broader host ranges or changed virulence. While there is currently scarce evidence for such genetic exchanges among phytoplasmas, the possibility of enhanced adaptability and evolutionary advancements in situations of mixed infections should not be overlooked.

Finally, mixed infections challenge current disease control strategies, as shown in Table 1, which often target a single phytoplasma strain or rely on symptom-based surveillance. Without accounting for co-infections, management efforts may be misdirected or insufficient. Integrated disease monitoring systems, incorporating multilocus molecular diagnostics and vector population studies, are essential for effectively identifying, predicting, and managing the spread of mixed phytoplasma infections.

## 3. Mixed Infections of Phytoplasmas and ‘Candidatus Liberibacter’ Species

Phytoplasmas and ‘*Ca.* Liberibacter’ species represent two critical groups of phloem-limited, unculturable, insect-transmitted plant pathogens that represent significant threats to global agricultural productivity. Both groups are disseminated by insect vectors possessing piercing–sucking mouthparts, comprising psyllids and leafhoppers, and are associated with various high-impact crop diseases [10,12,47,62,63,64]. Phytoplasmas are recognized for inducing witches’ broom, phyllody, and floral deformities in numerous host plants, while ‘*Ca.* Liberibacter’ species are primarily acknowledged for their involvement in diseases such as Huanglongbing (HLB) in citrus and zebra chip in potato [23,65,66,67]. The biological similarities observed between these pathogens, characterized by phloem localization, inability to be cultured, insect transmission, and symptomatic resemblance, render co-infection not only feasible but also probable, particularly in regions where susceptible hosts and proficient vectors coexist. Indeed, a growing body of research has documented mixed infections involving phytoplasmas and ‘*Ca.* Liberibacter’ species across various plant hosts and geographical locations [23,24,28,33,47,68]. These observations raise essential questions regarding pathogen interaction, symptom modulation, and their implications for disease diagnosis and management.

### 3.1. Co-Infection of Citrus Plants with Phytoplasmas and ‘Ca. L. asiaticus’

Among the most well-documented examples of mixed infection is the co-occurrence of phytoplasmas and ‘*Ca.* Liberibacter asiaticus’, the pathogen most associated with HLB, as shown in Table 2. HLB affects numerous citrus species, including *Citrus maxima*, *C. sinensis*, *C. aurantifolia*, and *C. × limon*, and represents one of the most devastating citrus diseases worldwide due to its rapid spread and lack of effective cure [64,66,67].

In China, particularly on Hainan Island, *C. maxima* trees have been observed exhibiting symptoms such as leaf yellowing, crinkling, and mottling, which closely resemble those of HLB. At the same time, the region is also endemic for several phytoplasma-associated diseases, suggesting the possibility of co-infection [69,70,71,72,73]. In a study performed by Yu et al. [33], the presence of two phytoplasma strains (belonging to the 16SrII and 16SrXXXII groups) along with ‘*Ca.* L. asiaticus’ was confirmed in symptomatic *C. maxima* plants using molecular diagnostics (Table 2). Of the 54 symptomatic samples tested, 22.2% were positive for 16SrII phytoplasma, 3.7% for 16SrXXXII, and 11.1% for ‘*Ca.* L. asiaticus’. Notably, 7.4% of the samples harbored mixed infections involving these phytoplasmas and ‘*Ca.* L. asiaticus’, supporting the hypothesis that these pathogens may co-occur naturally in field conditions. None of the tested asymptomatic plants harbored any of the pathogens, reinforcing the association between these plants and visible disease symptoms [33]. These findings highlight the complex etiology of citrus decline, where symptom severity and variability may result from pathogen interactions rather than single infections. Mixed infections may also contribute to diagnostic uncertainty, particularly because symptoms induced by phytoplasmas and ‘*Ca.* L. asiaticus’ are overlapping and difficult to distinguish visually.

**Table 2:** Mixed infections of phytoplasmas and ‘*Ca.* Liberibacter’ spp. in economically important citrus and carrot crops.

Phytoplasma/16Sr Groups	‘*Ca.* Liberibacter’ spp. Involving Mixed Infections	Hosts	Symptoms	Locations	References
16SrII-V	‘*Ca.* Liberibacter asiaticus’	*Citrus maxima*	Huanglongbing, including yellowing, crinkled leaves, mottled leaves	Hainan, China	[33]
16SrII	‘*Ca.* Liberibacter asiaticus’	*Citrus sinensis*	Huanglongbing	Iran	[68]
16SrI	‘*Ca.* Liberibacter asiaticus’	Citrus plants	Huanglongbing	Guangdong, China	[24]
16SrI	‘*Ca.* Liberibacter asiaticus’	Citrus plants	Huanglongbing	Mexico	[28]
16SrIX	‘*Ca.* Liberibacter asiaticus’	Citrus plants	Huanglongbing	Brazil	[23]
16SrI, 16SrII, 16SrIV, 16SrV, 16SrVII, 16SrX, 16SrXI, 16SrXII	‘*Ca.* Liberibacter asiaticus’	*Citrus latifolia*, *Citrus paradisi*, *Citrus sinensis*, *Citrus reticulata* × *C. sinensis*	Huanglongbing	Caribbean	[31]
16SrI	‘*Ca.* Liberibacter solanacearum’	Carrot	Leaf curling with yellow, twisting of petioles, stunted growth of shoots and roots	Spain	[47]

**Figure 1:**
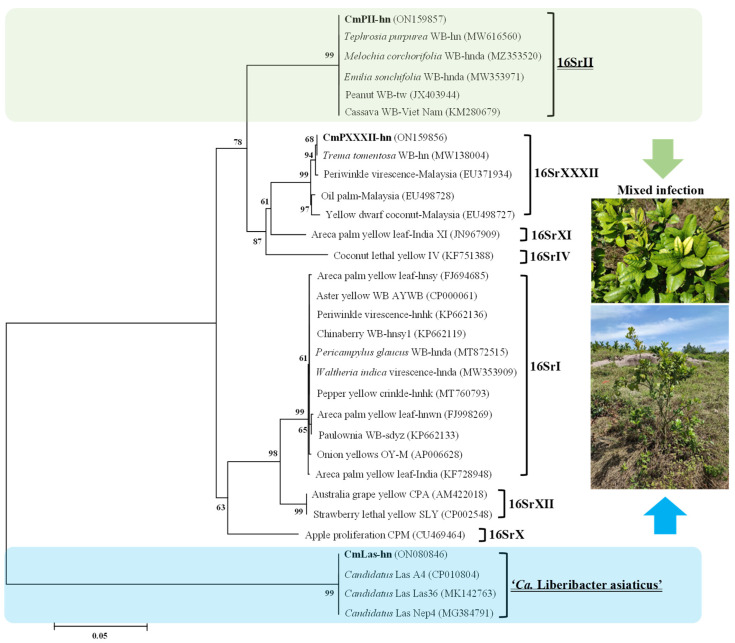
A phylogenetic tree constructed using the neighbor-joining method based on 16S rRNA gene sequences derived from various phytoplasma strains, including *C. maxima* phytoplasma CmPII-hn and CmPXXXII-hn strains, which belong to the 16SrII and 16SrXXXII groups, respectively, as well as the ‘*Ca.* Liberibacter asiaticus’ CmLas-hn strain, identified in *C. maxima* plants on Hainan Island, China. In this tree, the scale bar indicates inferred character-state changes, revealing the genetic diversity and evolutionary history of the analyzed strains. Branch lengths represent the number of inferred character-state changes, and the scale bar indicates genetic distance. Bootstrap values based on 1000 replicates are shown next to each branch, providing statistical support for the tree topology. The identification of strains from different clades co-infecting the same region suggests potential mixed infections, as highlighted by the accompanying images of symptomatic *C. maxima* plants. This analysis enhances our understanding of phylogenetic relationships between plant pathogens, which can impact plant health and management strategies [33].

Further genetic analysis by Yu et al. [33] revealed that the phytoplasmas detected in *C. maxima* belonged to the subgroups 16SrII-V and 16SrXXXII-D, both of which exhibited 100% sequence identity in the 16S rRNA gene with strains previously isolated from other Asian plant species, including *Tephrosia purpurea*, *Melochia corchorifolia*, and *Trema tomentosa*. Meanwhile, detected HLB strains exhibited complete sequence homology in 16S rRNA and β-operon genes with ‘*Ca.* L. asiaticus’ (reference strain A4, Accession No. CP010804), indicating strong genetic stability across geographic locations. Phylogenetic analysis (Figure 1) further confirmed these relationships, showing that the 16SrII and 16SrXXXII phytoplasmas cluster closely with reference strains from Southeast Asia, while ‘*Ca.* L. asiaticus’ strains form a distinct, well-supported clade aligned with global isolates. These findings illustrate that *C. maxima* serves as a susceptible host to multiple, genetically diverse pathogens, and that the co-occurrence of phytoplasmas and ‘*Ca.* L. asiaticus’ may reflect shared vector ecology or environmental drivers. Reports of similar mixed infections in citrus have also emerged from Iran, Brazil, Mexico, and the Caribbean [23,24,28,31,68], further confirming that this phenomenon is not geographically isolated but represents a global challenge for citrus production.

### 3.2. Mixed Infections in Non-Citrus Hosts: ‘Ca. L. solanacearum’ and Phytoplasmas

Beyond citrus, ‘*Ca.* L. solanacearum’, a close relative of ‘*Ca.* L. asiaticus’, has been identified as a major pathogen in solanaceous and apiaceous crops, including potato, carrot, and celery. First described in New Zealand in association with the zebra chip disease of potato [62], this pathogen has since been detected across Europe and North America, where it has emerged as a threat to both open-field and greenhouse-grown crops [63,65,74,75].

In a study by Satta et al. [47], carrot plants (cv. Córdoba) in Europe exhibited symptoms such as leaf curling, yellowing, twisted petioles, and stunted root and shoot growth. Molecular analysis revealed the presence of 16SrI group phytoplasmas co-infecting with ‘*Ca.* L. solanacearum’ (Table 2). The identification was confirmed through PCR amplification and RFLP analysis of the 16S rRNA gene, highlighting the diagnostic complexity associated with overlapping pathogen-induced symptoms in these mixed infections. These findings reinforce the idea that mixed infections involving phytoplasmas and ‘*Ca.* Liberibacter’ species are not confined to citrus but represent a wider agricultural concern, affecting diverse plant families in both tropical and temperate regions.

## 4. Mixed Infections of Phytoplasmas and Plant Viruses

Plant viruses play a crucial role in reducing crop yield and deteriorating quality worldwide, leading to a variety of economically damaging diseases that affect different plant species and environments. A significant number of these viruses are disseminated by insect vectors, such as leafhoppers, which are also responsible for transmitting phytoplasmas. In natural conditions, plants often face simultaneous attacks from both viruses and phytoplasmas (refer to Table 3). This overlap in the ecology of vectors and the susceptibility of hosts enables the possibility of mixed infections, which are gaining recognition as a crucial aspect of plant disease epidemiology [3,76]. The co-infection of plants by viruses and phytoplasmas introduces complexity due to the potential for either synergistic or additive effects, impacting disease severity, symptom complexity, and transmission dynamics. These interactions can heighten the physiological stress experienced by the host, accelerate disease progression, or make visual diagnosis more challenging. Symptoms such as leaf curling, mottling, yellowing, stunting, and reduced fruiting often present diagnostic difficulties as they are not easily linked to a single pathogen, thus complicating disease surveillance, diagnostics, and control efforts.

Recent studies have highlighted the natural co-infections of phytoplasmas and viruses across numerous plant species, resulting in effects that range from mild symptom changes to severe growth defects and even plant death. In India, co-infections of cucumber mosaic virus (CMV) with the 16SrII group phytoplasma have been associated with symptoms such as mosaic patterns, mottling, and reduced leaf size in *Solanum melongena* (eggplant) [77]. Similarly, in other solanaceous hosts, the concurrent infection of PVX and PVY with 16SrVI phytoplasma has been shown to cause comparable leaf distortion and chlorosis [78]. Furthermore, additional research has linked begomoviruses with the 16SrVI group phytoplasmas in solanaceous crops, resulting in symptoms such as mosaic, leaf curling, and bushy growth [79].

Sugarcane has also been identified as a host for dual infections, particularly involving the sugarcane yellow leaf virus (ScYLV) co-occurring with phytoplasmas from the 16SrI and 16SrXI groups. This combination results in generalized chlorosis, stunted internodes, and a decline in cane juice quality [80,81,82]. *Carica papaya*, a high-value fruit crop in tropical regions, may experience co-infection by the papaya ringspot virus (PRSV) and 16SrII phytoplasmas, a combination linked to decline syndromes in Cuba [83]. In the case of banana, co-infection with the banana bunchy top virus (BBTV), banana streak MY virus (BSMYV), and 16SrI-D subgroup phytoplasmas results in complicated symptomology, which encompasses dwarfing, leaf curling, and bunchy top symptoms [84].

In *Capsicum annuum* (chili pepper), co-infection with ageratum enation virus (AEV) and 16SrI phytoplasma has been demonstrated to result in significant yellowing, leaf deformation, and decreased fruit yield [49]. Similarly, in Mexico, the combination of begomovirus and 16SrIII group phytoplasma correlates with ‘yellow type’ diseases in pepper, exhibiting symptoms that closely resemble those observed in viral or phytoplasmal infections alone [85]. These studies highlight the widespread prevalence and crop specificity of virus–phytoplasma mixed infections, which negatively impact both annual and perennial crops in tropical and subtropical regions.

**Table 3:** Phytoplasma virus co-infections contribute to severe symptoms in horticultural and field crops.

Phytoplasma/16Sr Groups	Virus Involving Mixed Infections	Hosts	Symptoms	Locations	References
16SrII	Cucumber mosaic virus	*Solanum melongena*	mosaic, mottling, and small leaves	India	[77]
16SrVI	Potato virus X, potato virus Y	*Solanum melongena*	small leaves, leaf chlorosis, and malformations with mosaic mottling	India	[78]
16SrI-D	Banana bunchy top virus, banana streak MY virus	Banana plants	bunchy top, small leaves, leaf chlorosis, necrosis, and stunted growth	India	[84]
16SrXI	Sugarcane yellow leaf virus	Sugarcane	yellow and grassy shoot	India	[82]
16SrI	Sugarcane yellow leaf virus	Sugarcane	yellowing	Egypt	[81]
16SrI-A	Sugarcane yellow leaf virus	Sugarcane	yellow discoloration of the midrib	Cuba	[80]
16SrII	Papaya ringspot virus	*Carica papaya*	mosaic, yellowing, and crinkling	Cuba	[83]
16SrVI	Begomovirus	*Solanum melongena*	mosaic, small leaves	India	[79]
16SrVI	Tomato leaf curl virus	eggplant	little leaf, leaf curl	India	[86]
16SrI-B	Begomovirus	*Zinnia elegans*	leaf curling, and witches’ broom	India	[29]
16SrI-B	Ageratum enation virus	*Capsicum annum*	yellow, leaf curling, and small leaves	India	[49]
16SrVI-D	Tomato leaf curl virus	*Withania somnifera*	yellow and yellow-type little leaves	India	[49]
16SrIII	Begomovirus	Pepper		Mexico	[85]

## 5. Mixed Infections of Phytoplasmas with Spiroplasmas

Phytoplasmas and spiroplasmas are both members of the class Mollicutes and possess unique biological and ecological characteristics. They lack a rigid cell wall and are specifically found in the phloem tissues of plants. These pathogens rely on phloem-feeding insect vectors, like leafhoppers, for their transmission. Spiroplasmas are particularly notable for their helical shape and motility. Despite these differences, spiroplasmas share similarities with phytoplasmas, such as their localization within hosts and reliance on insect vectors for dispersal. These commonalities foster conditions that promote co-infection in natural environments. Historically, spiroplasmas have been associated with diseases, such as corn stunt (*Spiroplasma kunkelii*) and citrus stubborn disease (*Spiroplasma citri*), while phytoplasmas are linked to a broad range of disorders. The overlapping symptomatology and shared vector pathways suggest that mixed infections involving these two groups are not only possible but likely under field conditions (Table 4).

Several studies have confirmed natural co-infections of spiroplasmas and phytoplasmas in economically important crops (Table 4). In maize, Oliveira et al. [87] demonstrated that the leafhopper *Dalbulus maidis* could simultaneously transmit phytoplasmas and spiroplasmas, leading to co-infection in maize plants. Plants harboring both pathogens exhibited severe stunting, chlorosis, and leaf reddening, with more intense symptoms than those observed in single infections, indicating potential synergistic effects.

In palm species affected by lethal yellowing in Mexico, typically caused by phytoplasmas from the 16SrIV group, spiroplasmas were also detected. Using nested PCR and scanning electron microscopy, Lebsky et al. [88] confirmed the presence of both pathogens, suggesting that palm decline symptoms could result from dual infection and that the contribution of each pathogen might vary based on host and environmental conditions.

In Brazil, Galvão et al. [30] found that corn plants exhibiting stunting symptoms were infected with both phytoplasmas and spiroplasmas. Although the interaction between these two pathogens in co-infected maize plants remains unclear, the observed symptoms were notably more severe than those seen in singly infected plants, reinforcing the idea of enhanced pathogenicity through co-infection.

Perhaps the most complex case was reported by Salehi et al. [32] in sesame plants (*Sesamum indicum*) in Iran. The affected plants displayed classic symptoms of phyllody, virescence, and floral malformation. Molecular diagnostics revealed the co-presence of *Spiroplasma citri* and phytoplasmas belonging to four different subgroups: 16SrII-C, 16SrII-D, 16SrVI-A, and 16SrIX-C. The identification of multiple pathogens in a single host exemplifies the diverse and dynamic nature of plant-pathogen interactions in the field.

**Table 4:** Mixed infections of phytoplasmas and other pathogens, including spiroplasma, fungi, and other phloem-restricted bacteria.

Phytoplasmas/16Sr Groups	Pathogens Involving Mixed Infections		Hosts	Symptoms	Locations	References
	Kinds	Names				
Phytoplasma	Spiroplasma	Spiroplasma	*Dalbulus maidis*	ND	Brazil	[87]
16SrIV		Spiroplasma	Palm species	yellowing	Mexico	[88]
Phytoplasma		Spiroplasma	Corn	stunting	Brazil	[30]
16SrII-C, 16SrII-D, 16SrVI-A, 16SrIX-C		*Spiroplasma citri*	Sesame	phyllody	Iran	[32]
Stolbur phytoplasma	‘*Ca.* Phloemobacter’ species	‘*Ca.* Phloemobacter fragariae’	Sugar beet	‘basses richesses’ syndrome	Europe	[89]
Stolbur phytoplasma		‘*Ca.* Phloemobacter fragariae’	Strawberry	marginal chlorosis	France	[90]
‘*Ca.* Phytoplasma solani’	‘*Ca.* Arsenophonus’ species	‘*Ca.* Arsenophonus phytopathogenicus’	Sugar beet	‘basses richesses’ syndrome	Germany	[35]
‘*Ca.* Phytoplasma solani’		‘*Ca.* Arsenophonus phytopathogenicus’	Beet, potato plants	‘basses richesses’ syndrome	Germany	[34]
‘*Ca.* Phytoplasma solani’		‘*Ca.* Arsenophonus phytopathogenicus’	*Pentastiridius leporinus*	ND	Germany	[34]
16SrI-X, 16SrII-N	Rickettsia	Rickettsia	Papaya	bunchy top	Cuba	[27]
‘*Ca.* Phytoplasma solani’	Fungus	*Macrophomina phaseolina*	Sugar beet	rubbery taproot, charcoal root rot	Central Europe	[35]

ND represents not determined.

## 6. Mixed Infections of Phytoplasmas with Other Unculturable Plant Pathogens

Phytoplasmas, alongside well-documented co-infections involving ‘*Ca.* Liberibacter’ species, viruses, and spiroplasmas, are also associated with other unculturable, phloem-restricted bacterial pathogens, notably from the genera ‘*Ca.* Phloemobacter’ and ‘*Ca.* Arsenophonus’ (Table 4). These pathogens, similar to phytoplasmas, are transmitted by insect vectors and reside in the phloem of host plants, resulting in overlapping symptoms and frequent diagnostic confusion. Despite being studied less than other phytopathogens, ‘*Ca.* Phloemobacter fragariae’ and ‘*Ca.* Arsenophonus phytopathogenicus’ have been linked to significant crop diseases. Their co-infection with phytoplasmas complicates the understanding of disease etiology, symptom development, and host responses. This highlights the necessity for more comprehensive studies on mixed infection systems in vascular plants.

One of the earliest documented cases of co-infection involving a phytoplasma and ‘*Ca.* Phloemobacter fragariae’ was linked to the ‘basses richesses’ syndrome of sugar beet, first described in France in 1991. This disease, resulting in reduced sugar content in beets, was associated with a mixed infection involving a stolbur phytoplasma (16SrXII group) and ‘*Ca.* Phloemobacter fragariae’ [89]. Although both pathogens colonize phloem tissues and cause systemic yellowing and decline, subsequent histological analyses revealed distinct cellular effects. Phytoplasmas were related to cell necrosis and lignification, while ‘*Ca.* Phloemobacter fragariae’ also stimulated phenolic compound deposition within the phloem lumen, indicating pathogen-specific alterations in host vascular physiology.

A similar co-infection was reported in marginal chlorosis of strawberries, a disease that has impacted strawberry cultivation in France for over three decades. This syndrome was attributed to a color-type phytoplasma in conjunction with ‘*Ca.* Phloemobacter fragariae’ [90]. While both pathogens contributed to leaf chlorosis and marginal necrosis, only molecular diagnostics could confirm the presence of both agents, as symptom-based diagnosis proved unreliable due to the indistinguishable visual presentation of single and mixed infections.

More recently, Behrmann et al. [34] and Duduk et al. [35] reported the co-infection of sugar beet and potato crops in Germany with two phloem-restricted bacterial pathogens: the stolbur phytoplasma ‘*Ca.* Phytoplasma solani’ and the γ-proteobacterium ‘*Ca.* Arsenophonus phytopathogenicus’. These mixed infections were associated with stunting, chlorosis, and vascular collapse, symptoms commonly linked to each pathogen individually. However, the presence of both organisms in the same plant tissues complicates attribution and raises important questions regarding their respective roles and interactions during disease development.

Although phytoplasmas, ‘*Ca.* Phloemobacter fragariae’, and ‘*Ca.* Arsenophonus phytopathogenicus often produces similar visual symptoms, but the histological and physiological consequences of infection may differ. For instance, phytoplasma infection has been linked to cell wall thickening, necrosis, and lignification, processes that interfere with nutrient flow and lead to systemic symptoms, such as leaf curling and chlorosis. In contrast, ‘*Ca.* Phloemobacter fragariae’ infections have been shown to induce similar effects but are additionally associated with abnormal accumulations of phenolic compounds in phloem tissues, potentially influencing host defense signaling and vascular blockage [89]. These subtle yet significant differences highlight the importance of detailed anatomical and molecular investigations in cases where phloem-restricted pathogens co-infect the same host. The challenge is accurately attributing symptoms and disease severity to specific pathogens, particularly in the presence of mixed infections. Conventional diagnostic tools often focus on single pathogens, overlooking the presence of others, which can lead to incomplete diagnoses and suboptimal management strategies.

Furthermore, Acosta et al. [27] found that papaya could be co-infected by phytoplasma and rickettsia (Table 4). In Cuba, the pathogens 16SrI ‘*Ca.* Phytoplasma asteris’, 16SrII ‘*Ca.* Phytoplasma aurantifolia’ and rickettsia were simultaneously detected in two different papaya diseases, Bunchy Top Symptom (BTS) and Papaya Bunchy Top (PBT). The results obtained by Acosta et al. [27] confirmed that phytoplasmas were consistently associated with both BTS and PBT symptoms, and that mixed infections of the pathogens phytoplasmas and rickettsia were identified in either BTS- or PBT-affected papaya fields, which implied that new challenges would be faced in the epidemiological monitoring and effective prevention and control of these diseases.

## 7. Mixed Infections of Phytoplasmas with Fungal Pathogens

Increasing evidence has demonstrated that phytoplasmas can also co-infect plants with eukaryotic pathogens, particularly fungi [35] (Table 4). These cross-kingdom co-infections, although relatively underexplored, have the potential to compound disease severity, alter symptom development, and influence the outcome of pathogen–host interactions in agriculturally important crops. Fungi are among the most widespread and damaging plant pathogens globally, capable of infecting a wide range of hosts and tissues. When fungal pathogens co-occur with phytoplasmas, plants may experience multiple, interacting biotic stresses that amplify disease progression and increase susceptibility to secondary infections. Understanding these co-infections is critical for developing holistic plant health strategies that address complex, multi-pathogen systems.

Recent research on sugar beet (*Beta vulgaris*) provides a great example of phytoplasma–fungal co-infection. Duduk et al. [35] reported that sugar beet plants can be infected simultaneously by two pathogens: the stolbur phytoplasma, ‘*Ca.* P. solani’, and the soilborne fungal pathogen, *Macrophomina phaseolina*. Each of these pathogens causes distinct diseases. ‘*Ca.* P. solani’ leads to rubbery taproot syndrome, while *M. phaseolina* is responsible for charcoal root rot. The rubbery taproot, resulting from phytoplasma infection, softens the structural integrity of the root. This impairment makes the plant increasingly vulnerable to colonization by opportunistic soil fungi. When *M. phaseolina* subsequently infects these sugar beet plants, it triggers symptoms of charcoal root rot, which include blackened root tissues, vascular necrosis, and tissue collapse. This dual infection can lead to greater yield loss and hastened plant decline compared to single infections, clearly demonstrating a synergistic effect between the two pathogens.

The co-infection of sugar beet with ‘*Ca.* P. solani’ and *M. phaseolina* highlights the need for integrated diagnostic protocols capable of detecting both prokaryotic and eukaryotic pathogens. Traditional methods often target either fungal or bacterial agents separately, resulting in partial or inaccurate diagnoses in cases of complex infections. Molecular tools, such as multiplex PCR, qPCR, CRISPR-based diagnostics, and metagenomic sequencing, are increasingly necessary for the comprehensive detection of mixed infections involving pathogens from different biological kingdoms.

From a disease management perspective, cross-kingdom co-infections introduce additional complications. Control strategies focused solely on fungal pathogens, such as fungicides or soil treatments, may do little to address the underlying vascular disruption caused by phytoplasmas. Conversely, vector control strategies aimed at reducing phytoplasma transmission will not lessen the impact of an already established fungal infection. Furthermore, co-infections may influence host susceptibility, as an initial phytoplasma infection could weaken host defenses, creating pathways or physiological niches for fungal colonization.

Although phytoplasma–fungal co-infections have not yet been widely reported across crops, the sugar beet case study indicates that such interactions may be more common than currently appreciated, especially in stress-prone agroecosystems. The convergence of root-infecting fungi and phloem-limited bacteria in susceptible hosts could lead to novel disease syndromes that are difficult to classify or manage using traditional, pathogen-specific frameworks.

There is a pressing need for further research into the mechanisms of cross-kingdom interactions between phytoplasmas and fungi. Key questions include whether phytoplasma infection suppresses plant immunity in ways that facilitate fungal colonization or whether fungal infection affects vascular function in ways that enhance phytoplasma survival and spread. Understanding these dynamics will be critical for developing more effective cross-protective disease control strategies, especially in high-value crops that are susceptible to multiple soilborne and phloem-limited pathogens.

## 8. Diversity of Phytoplasmas and Other Pathogens and Their Influence on Mixed Infections

### 8.1. Geographic Hotspots and the Ecological Basis of Mixed Infections

Mixed infections are more likely to occur in regions where high pathogen diversity and overlapping host ranges coincide. In such ecosystems, plants are frequently exposed to multiple phytoplasma strains from different 16Sr groups as well as other pathogens, creating conditions conducive to co-infection. Hainan Province, a tropical island in southern China, serves as a prime example of such a hotspot. Numerous phytoplasma strains and other phloem-restricted pathogens have been identified in symptomatic plants, often with overlapping symptoms and shared insect vectors [33,72,91,92,93].

In Hainan, Catharanthus roseus (periwinkle) exhibits different symptoms depending on the infecting phytoplasma group. Infection by the 16SrI-B group causes little leaf, virescence, and phyllody, whereas infection by the 16SrV group phytoplasmas leads to yellowing. Similarly, *Melochia corchorifolia* expresses phyllody when infected with 16SrI-B phytoplasma but develops witches’ broom under 16SrII-A infection. In *Carica papaya*, symptoms such as leaf malformation, yellowing, and little leaf have been linked to infection by phytoplasmas of the 16SrI-AP, 16SrI-B, and 16SrII-U groups, respectively. *Praxelis clematidea* presents witches’ broom and phyllody when infected with 16SrII-A and 16SrII-V groups, respectively. However, in some hosts, such as *Hevea brasiliensis* (rubber tree), infections by either 16SrI or 16SrII phytoplasmas, whether alone or in combination, consistently produce stem fasciation [94].

These examples demonstrate that the same plant species can exhibit distinct symptoms when infected by different phytoplasmas, and conversely, different phytoplasmas can induce similar symptoms in the same host. This diversity complicates diagnosis and highlights the need for molecular tools to differentiate the causal agents of phytoplasma-related diseases accurately.

### 8.2. Pathogen Diversity Across Regions and Its Role in Disease Expression

Pathogen diversity across regions also contributes to variability in symptom expression and the potential for co-infection. In China, for example, sugarcane white leaf disease, one of the most economically damaging diseases of sugarcane, is caused by different phytoplasma groups in different provinces: 16SrII in Hainan and 16SrXI in Yunnan [95]. This regional variation emphasizes that pathogen identity may not correlate strictly with symptomatology, especially when the same disease manifests under different climatic or ecological conditions. An illustrative example is *Citrus maxima* in Hainan, which shows yellowing and mottled leaves caused by co-infection with three distinct pathogens: 16SrII-V phytoplasma, 16SrXXXII-D phytoplasma, and ‘*Ca.* L. asiaticus’ [33]. These complex infections highlight the necessity for a multi-pathogen diagnostic strategy, especially in areas with considerable pathogen diversity and overlapping hosts.

### 8.3. Reservoir Hosts and Their Role in Pathogen Spread

Mixed infections are not exclusive to cultivated crops. Weeds and wild plants act as significant reservoirs for pathogenic organisms, facilitating both the persistence and spread of phytoplasmas and other pathogens. The role of reservoir plants is especially important in mixed farming systems or marginal lands surrounding cultivated areas. Richardia scabra, a common weed in sugarcane fields in China, was found to harbor a phytoplasma strain closely related (99.7% 16S rRNA sequence identity) to the sugarcane white leaf phytoplasma [91]. These findings suggest that weeds can sustain phytoplasma populations during off-seasons or periods of vector dormancy, serving as crucial sources for reinfection in subsequent planting cycles.

Additionally, the high sequence homology (>99%) among phytoplasma strains infecting cassava in China, Vietnam, and Thailand suggests potential regional transmission or shared reservoir hosts. Similarly, phytoplasmas infecting *Piper nigrum* (black pepper) have been shown to share over 99.8% sequence identity with those infecting several other plant species on Hainan Island, including *Carica papaya*, *Melia azedarach*, *Capsicum annuum*, *Pericampylus glaucus*, *Catharanthus roseus*, and *Malvastrum coromandelianum* [91]. These findings reinforce the hypothesis that highly homologous phytoplasmas can spread among phylogenetically and ecologically diverse hosts, especially in regions where multiple susceptible species coexist.

### 8.4. Regional Expansion of 16SrXXXII Phytoplasmas and Co-Infection Risk

One of the most rapidly expanding phytoplasma groups in Asia is the 16SrXXXII group, which has a known distribution in East and Southeast Asia. Subgroups 16SrXXXII-A, -B, and -C have been described in Malaysia [96,97], while subgroups 16SrXXXII-D, -E, and -F have been identified in China, South Korea, and Japan [33,36,69,92,93,98,99]. Notably, this group is responsible for a range of plant diseases across diverse hosts, including *Trema tomentosa* witches’ broom, *Citrus maxima* yellow leaf, *Camptotheca acuminata* witches’ broom, and *Elaeocarpus sylvestris* decline in China; coconut yellow dwarf and periwinkle phyllody in Malaysia; and *Elaeocarpus zollingeri* yellows in Japan.

A confirmed case of co-infection between 16SrXXXII and 16SrI group phytoplasmas has been reported in Elaeocarpus sylvestris trees affected by decline syndrome on Jeju Island, South Korea [36]. However, no other confirmed co-infections involving 16SrXXXII and other groups have been reported to date. Given the extensive host range and broad geographic distribution of this group, the potential for additional mixed infections remains high, especially in regions where co-host species overlap within a limited area [33,91].

### 8.5. Implications for Quarantine, Surveillance, and Disease Prevention

The evidence of mixed infections involving multiple phytoplasma groups and other pathogens in a geographically restricted region with diverse plant species raises critical concerns for phytosanitary management and biosecurity. Host plants, such as *Areca catechu* and *Citrus maxima*, which are prone to infection by multiple phytoplasmas and Liberibacter species, should be subject to simultaneous inspection and quarantine during cross-border transportation and trade. Comprehensive surveillance systems incorporating molecular diagnostics, vector monitoring, and host range studies are essential for preventing the spread of complex pathogen consortia. Targeted identification of reservoir hosts, including asymptomatic wild plants and weeds, will further strengthen early warning systems and guide integrated pest and disease management strategies.

## 9. Prospects for Monitoring and Managing Phytoplasma-Associated Mixed Infections

Phytoplasmas are unculturable, phloem-limited bacterial pathogens that present unique diagnostic challenges. The presence of mixed infections involving other phytoplasma groups, bacteria, and viruses, or even fungal pathogens, exacerbates the difficulties encountered in current diagnostic efforts. This highlights the need for comprehensive diagnostic protocols that include phytoplasmas in their detection panels, especially in areas where these organisms coexist with other pathogens. The failure to detect phytoplasmas or co-infected pathogens in instances of mixed infections may lead to misdiagnosis and inadequate disease management. Consequently, the identification of phytoplasmas and concurrent pathogens should be an integral component of disease surveillance programs, especially in tropical and subtropical areas characterized by a high diversity of vectors and considerable overlap of pathogens.

The interactions between phytoplasmas and other co-infecting pathogens within the same host remain an important yet understudied area of plant pathology. In mixed infections, phytoplasmas may act as either primary or secondary pathogens, and distinguishing their roles in disease expression from those of other microbes can be challenging. For example, in *Citrus maxima*, simultaneous infection by phytoplasmas and ‘*Ca.* L. asiaticus’ was confirmed, but their respective contributions to the observed symptoms, such as yellowing and leaf mottling, are not yet fully understood [33]. It remains unclear whether these pathogens compete for phloem resources, suppress or enhance each other’s activity, or co-modulate host responses in ways that exacerbate disease severity. Understanding these dynamics requires targeted research into molecular crosstalk, plant immune modulation, and vector behavior in mixed infection contexts. Such studies would provide critical insights into how phytoplasmas interact with other pathogens and how these interactions affect disease development, transmission, and epidemiology.

Mixed infections significantly complicate disease management strategies. Since phytoplasmas cannot be cultured in vitro and their detection relies on molecular tools, they may easily be overlooked in standard disease management protocols. Furthermore, they often share insect vectors with other pathogens or may be transmitted independently by a distinct group of insects. For instance, leafhoppers are primary vectors of phytoplasmas, while psyllids transmit Liberibacter species [17,64,66,67,100]. Therefore, vector control strategies must be carefully designed to consider the full spectrum of pathogen–vector relationships within the local pathosystem.

In addition to vector management, phytoplasma–host interactions can influence susceptibility to co-infection by other pathogens. Phytoplasmas often disrupt host development by secreting effector proteins, altering hormone balances, and weakening defense responses [22,101]. These disruptions may create physiological conditions favorable to subsequent colonization by viruses, fungi, or other bacteria. Recognizing phytoplasma infections as a potential predisposing factor for other diseases could change how early intervention strategies are prioritized in integrated pest and disease management programs.

Breeding for resistance to phytoplasmas has historically been limited due to the difficulties in detection and the absence of reliable artificial inoculation methods. However, with advances in molecular diagnostics and increased awareness of phytoplasma diversity, it has become clear that many important crops, such as citrus, papaya, sugarcane, sesame, and cassava, are frequently infected by multiple phytoplasma groups, sometimes concurrently [21,27,31,36,38,82,91]. Traditional breeding strategies that focus on resistance to a single phytoplasma strain or subgroup are unlikely to be effective in field conditions, where mixed infections by different 16Sr groups are common. Additionally, resistance conferred against one phytoplasma group may not protect against others, or co-infecting pathogens, like ‘*Ca.* L. asiaticus’, or plant viruses. Therefore, resistance breeding must account for the complexity of co-infection, including natural field exposure to multiple pathogens. Multi-year and multi-location trials should include molecular screening for phytoplasmas to ensure that candidate lines exhibit durable resistance across pathogen variants. Moreover, breeding programs should prioritize the development of cultivars with broad-spectrum resistance, potentially by targeting common host pathways hijacked by phytoplasma effectors or by enhancing basal immunity in vascular tissues. The integration of genomics, transcriptomics, and genome editing tools may further facilitate the development of varieties resistant to complex infection pressures.

To enhance the early detection and management of phytoplasma-associated mixed infections, plant health surveillance systems must adapt to incorporate routine molecular diagnostics for various phytoplasma groups, along with vector monitoring and tracking of pathogen populations. High-throughput platforms, such as multiplex PCR and metagenomic sequencing, can significantly improve the ability to detect mixed infections, even in asymptomatic plants or during the early stages of disease development. Regional surveillance should also include reservoir host plants, such as weeds and ornamentals, that may harbor phytoplasmas and serve as sources of reinfection. Given the mobility of insect vectors and the role of asymptomatic carriers, landscape-level disease modeling is crucial for understanding how phytoplasmas disseminate through agricultural and ecological systems.

Quarantine measures must also reflect this complexity. In regions like Southeast Asia, where phytoplasmas from multiple 16Sr groups coexist, plant materials and propagation products should undergo simultaneous testing for all locally known phytoplasma groups, particularly for high-risk hosts such as *Citrus maxima*, *Areca catechu*, and *Elaeocarpus sylvestris*.

## 10. Conclusions

Mixed infections involving phytoplasmas and other plant pathogens represent a complex and emerging area of research in plant pathology. Far from being isolated events, such co-infections are increasingly recognized as common occurrences in diverse agroecosystems, especially in tropical and subtropical regions where phytoplasmas from multiple 16Sr groups coexist with bacteria like ‘*Ca.* Liberibacter’ spp., viruses, fungi, and spiroplasmas. These interactions complicate disease diagnosis, obscure symptom specificity, and challenge the efficacy of pathogen-specific control strategies.

Phytoplasmas, due to their phloem-limited lifestyle, host manipulation capabilities, and reliance on insect vectors, can significantly alter plant physiology and immune responses, potentially predisposing hosts to secondary infections. However, the ecological and molecular dynamics of phytoplasma co-infections, whether synergistic, antagonistic, or neutral, remain poorly understood. Clarifying these interactions is crucial for accurately identifying causal agents, predicting disease outcomes, and developing integrated management approaches.

To manage these complex pathosystems effectively, future efforts must focus on developing multi-pathogen diagnostic tools, expanding vector surveillance, and integrating resistance screening into breeding programs under natural co-infection conditions. A shift towards system-level plant health strategies, including phytoplasma-related diagnostics, epidemiology, and biosecurity, is essential. Understanding and addressing phytoplasma-associated mixed infections from this perspective will be critical for safeguarding crop productivity and enhancing sustainable plant disease management in an increasingly interconnected and pathogen-rich agricultural landscape.
